# Supplementary material for: Complex‐centric proteome profiling by SEC‐SWATH‐MS
Source: Mol Syst Biol. 2019 Jan 14;15(1):e8438. doi: 10.15252/msb.20188438 (PMC6346213; doi:10.15252/msb.20188438)
Supplement: Supplementary file 7 — Dataset EV6 [file MSB-15-e8438-s007.zip › feature_plots_bioplex/Q13227.pdf]

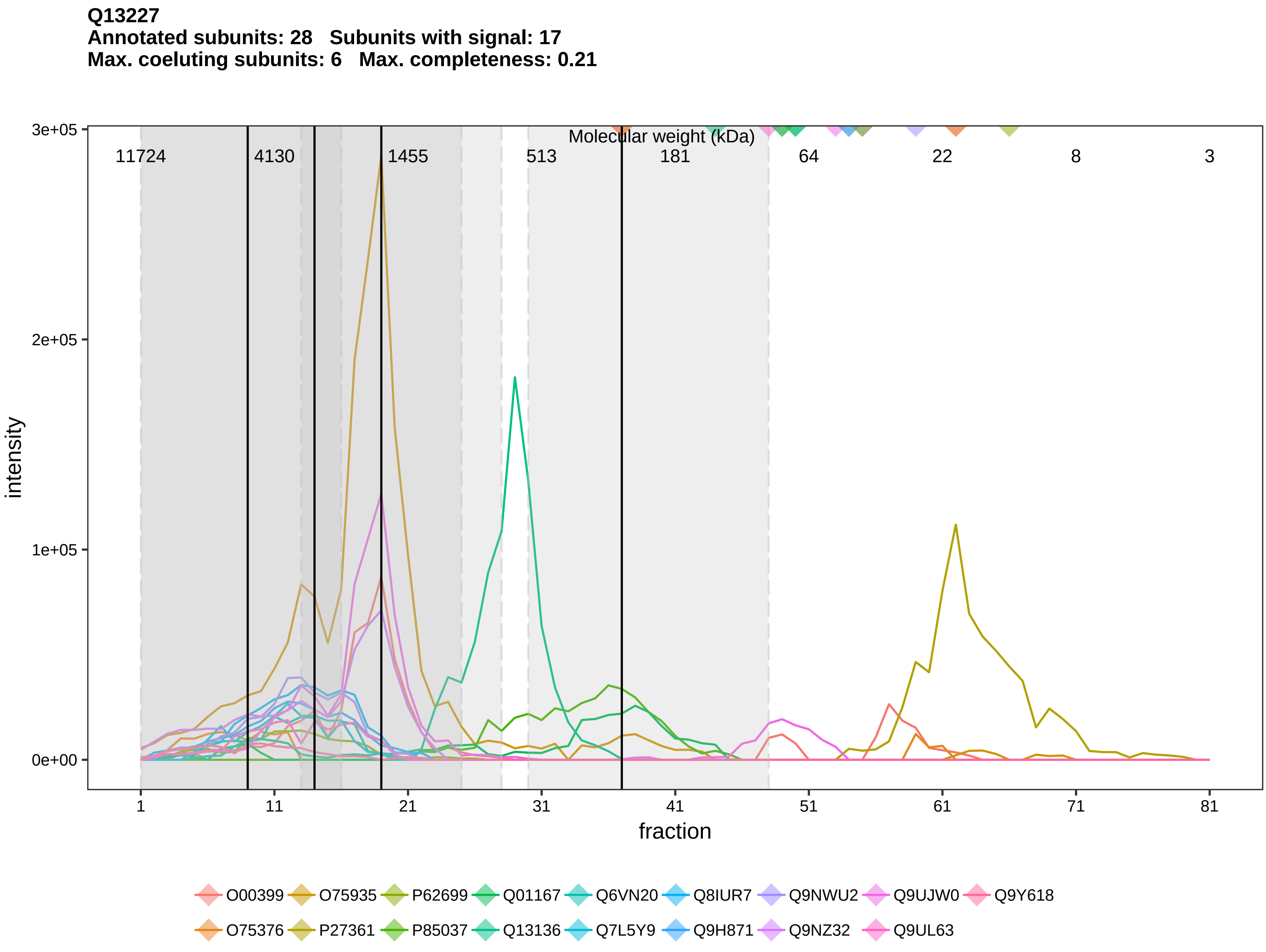

Q13227
Annotated subunits: 28 Subunits with signal: 17
Max. coeluting subunits: 6 Max. completeness: 0.21
Molecular weight (kDa)
11724
4130
1455
513
181
64
22
8
3
3e+05
2e+05
1e+05
0e+00
intensity
1
11
21
31
41
51
61
71
81
fraction
O00399
O75935
P62699
Q01167
Q6VN20
Q8IUR7
Q9NWU2
Q9UJW0
Q9Y618
O75376
P27361
P85037
Q13136
Q7L5Y9
Q9H871
Q9NZ32
Q9UL63
